# 8-Nitrotryptanthrin inhibits colorectal cancer progression via TGF-β/SMAD and PI3K/AKT/mTOR pathways

**DOI:** 10.3389/fphar.2025.1576673

**Published:** 2025-08-22

**Authors:** Cheng-Yu Sun, Kai-Ping Cong, Dan-Dan Zhao, En-Guo Fan, Ming-Quan Guo, Zheng-Guo Zhang

**Affiliations:** ^1^ Department of Colorectal Surgery, The Affiliated Xuzhou Clinical College of Xuzhou Medical University, Xuzhou, Jiangsu, China; ^2^ Laboratory of Advanced Theranostic Materials and Technology, Ningbo Institute of Materials Technology and Engineering, Chinese Academy of Sciences, Ningbo, China; ^3^ Ningbo Key Laboratory of Biomedical Imaging Probe Materials and Technology, Zhejiang International Cooperation Base of Biomedical Materials Technology and Application, Ningbo Cixi Institute of Biomedical Engineering, Ningbo, China; ^4^ School of Life Science, Jiangsu Normal University, Xuzhou, Jiangsu, China; ^5^ Peking Union Medical College, Beijing, China

**Keywords:** colorectal neoplasms, 8-nitrotryptanthrin, signal transduction, transforming growth factor beta, phosphatidylinositol 3-kinases

## Abstract

**Objective:**

To investigate the anticancer effects and underlying mechanisms of 8-nitrotryptanthrin (8-Nitrotryp) against colorectal cancer (CRC).

**Methods:**

The effects of 8-Nitrotryp on proliferation, colony formation, and migration were evaluated in HCT116 and SW480 cells, with comparisons to its parent compound tryptanthrin (Tryp). Mitochondrial membrane potential (MMP) was assessed using JC-1 staining, and early apoptosis was analyzed by flow cytometry. Proteomic analysis and Western blotting were employed to examine the modulation of the phosphatidylinositol 3-kinase (PI3K)/protein kinase B (AKT)/mechanistic target of the rapamycin (mTOR) pathway and transforming growth factor-β (TGF-β)/Sma- and Mad-related proteins (SMAD) signaling pathways, as well as epithelial–mesenchymal transition (EMT).

**Results:**

8-Nitrotryp significantly inhibited proliferation of HCT116 (IC_50_ = 0.81–1.08 μM; *P* < 0.001) and SW480 cells (IC_50_ = 0.76–1.59 μM; *P* < 0.001), suppressed colony formation of HCT116 (*P* < 0.001 at 1 μM) and SW480 cells (*P* < 0.001 at 2 μM), and inhibited migration in a dose-dependent manner (0.5–2 μM), demonstrating greater potency than Tryp. It also suppressed MMP and induced early apoptosis in HCT116 (*P* < 0.001 at 1 μM) and SW480 cells (*P* < 0.001 at 0.5 μM). Proteomic analysis and Western blotting revealed that 8-Nitrotryp downregulated PI3K expression, inhibited the phosphorylation of AKT and mTOR, and reduced TGF-β1-induced SMAD2 phosphorylation. Additionally, 8-Nitrotryp suppressed the EMT process.

**Conclusion:**

8-Nitrotryp inhibits CRC progression by modulating the TGF-β/SMAD and PI3K/AKT/mTOR pathways, highlighting its potential as a multi-target therapeutic agent for CRC and warranting its further investigation.

**Novelty and Impact:**

CRC is a global health challenge with limited treatments for advanced stages. This study provides the first evidence of 8-Nitrotryp’s antitumor efficacy in CRC, demonstrating its dual inhibitory activity on the TGF-β/SMAD and PI3K/AKT/mTOR pathways. Compared to Tryp, 8-Nitrotryp exhibits markedly enhanced potency, with lower IC_50_ values due to the introduction of a nitro group. Furthermore, the suppression of EMT is mechanistically linked to TGF-β/SMAD pathway inhibition. These findings suggest 8-Nitrotryp’s potential as a novel therapeutic for CRC.

## 1 Introduction

Colorectal cancer (CRC) ranks among the most prevalent malignant tumors that affect the digestive system. Its incidence is rising, with rapid progression and low overall survival rates. Worldwide, it is the third most frequently diagnosed cancer and the second major cause of cancer-related mortality, representing a serious challenge to public health. In 2020, an estimated 1.93 million new cases of CRC were reported worldwide, along with 935,000 associated deaths (Sung et al., 2021). It is estimated that the burden of this disease will increase by approximately 60% by 2030, reaching over 2.2 million new cases and more than 1.1 million deaths (Arnold et al., 2017). Approximately 20%–25% of CRC patients present with distant metastases at initial diagnosis (Smolenschi et al., 2021). For CRC, the main treatment options currently include radical surgical resection, chemotherapy, radiotherapy, and targeted drug therapy. However, challenges such as patient tolerance and tumor recurrence remain unresolved. Thus, CRC prevention and control represent a significant challenge. It is therefore crucial to discover new and effective treatments for CRC (Saitoh, 2015).

CRC exhibits significant molecular heterogeneity, commonly classified into consensus molecular subtypes (CMS1-4) and microsatellite instability (MSI) status (MSI-high vs. microsatellite stable, MSS). These subtypes demonstrate distinct clinical behaviors, prognoses, and treatment responses. CMS4 (mesenchymal) tumors are frequently associated with transforming growth factor-β (TGF-β) pathway activation, stromal invasion, and poor prognosis (Sung et al., 2021). The TGF-β/Sma- and Mad-related proteins (SMAD) and phosphatidylinositol 3-kinase (PI3K)/protein kinase B (AKT)/mechanistic target of rapamycin (mTOR) pathways are frequently dysregulated across subtypes, with TGF-β signaling particularly prominent in mesenchymal/CMS4 tumors and PI3K/AKT activation often driven by mutations in Kirsten RAt Sarcoma viral oncogene homolog (KRAS), B-Raf Alpha Fusion (BRAF), or PI3K catalytic subunit alpha (PIK3CA), prevalent in specific subtypes like CMS3 (metabolic) and CMS2 (canonical) (Saitoh, 2015). The TGF-β1/SMAD pathway is a key signaling route implicated in the initiation and progression of cancer. It regulates several physiological processes, including cancer cell growth and epithelial–mesenchymal transition (EMT). This pathway also influences various biological functions of tumor cells, such as proliferation, differentiation, and cell motility (Bierie and Moses, 2006). TGF-β1 binds to the TGF-β type II receptor, forming an active complex that phosphorylates and activates the TGF-β type I receptor. This activation triggers the phosphorylation of transcription factors SMAD2/3, which then bind to the mediator SMAD4 and translocate to the nucleus, establishing the TGF-β/SMAD signaling pathway (Zhang, 2017). The TGF-β/SMAD axis also exhibits subtype-specific functions in CRC, particularly in microsatellite instability-high (MSI-H) models such as HCT116. Aberrant TGF-β signaling is a hallmark of aggressive CRC subtypes. In these cells, autocrine TGF-β signaling persists through the type I TGF-β receptor kinase (TβRI) despite type II TGF-β receptor kinase (TβRII) deficiency, leading to SMAD2 activation and promoting invasive behavior independently of exogenous TGF-β (Morikawa et al., 2016). The phosphatidylinositol 3-kinase/protein kinase B/mammalian target of the rapamycin (PI3K/AKT/mTOR) pathway is another critical signaling cascade involved in cell proliferation, survival, and metabolism (Lamouille et al., 2014). The dysregulation of this cellular pathway, often driven by mutations in KRAS or PIK3CA common in MSS tumors like SW480, is frequently observed in various cancers including CRC, making it an attractive target for cancer therapy.

Natural products have historically served as a valuable resource for drug discovery, with numerous compounds exhibiting strong anticancer properties (Sun et al., 2023). Tryptanthrin (Tryp), chemically known as indolo[2,1-b]quinazoline-6,12-dione, is an indoloquinazoline alkaloid. Tryp is primarily derived from indigo-producing plants, such as *Isatis indigotica*, *Strobilanthes cusia*, and *Polygonum tinctorium*. Additionally, it can be isolated through the cultivation of *Yarrowia lipolytica*. Various synthetic methods for Tryp have also been developed. Tryp and its derivatives demonstrate broad anticancer effects, modulating inflammatory responses, inducing apoptosis, and suppressing proliferation across tumor models (Laurindo et al., 2023; Lin et al., 2021; Chang et al., 2019; Hsuan et al., 2009). They also show the potential to reverse chemoresistance, suggesting utility as chemoadjuvants (Zeng et al., 2021; Pathania et al., 2014; Shankar et al., 2020; Gao et al., 2021). Notably, nitro-substituted derivatives like 8-nitrotryptanthrin exhibit significantly enhanced potency compared to the parent compound (Zou et al., 2023).

Given the enhanced activity of 8-Nitrotryp in other cancers and the critical role of TGF-β/SMAD and PI3K/AKT/mTOR signaling in CRC progression across molecular subtypes, we hypothesized that 8-Nitrotryp exerts potent antitumor effects against CRC by inhibiting these key oncogenic pathways and suppressing associated processes like EMT.

To investigate the effects of 8-Nitrotryp across diverse CRC biology, we selected two well-characterized cell lines representing distinct molecular subtypes: HCT116 (MSI-H, *KRAS* ^G13D^, TP53 wild-type) exhibits rapid proliferation and represents MSI-H tumors with defective mismatch repair; SW480 (MSS, *KRAS* ^G12V^, TP53 mutant) displays strong migratory and invasive behavior, characteristic of aggressive MSS subtypes often associated with KRAS mutations and EMT. By employing both models, a comprehensive evaluation of the antitumor activity of Tryp and 8-Nitrotryp across proliferative and metastatic phenotypes is enabled. Proteomic analysis was employed to investigate 8-nitrotryp’s anti-CRC mechanisms, specifically those targeting pathways (TGF-β/SMAD, PI3K/AKT/mTOR) known to be critical drivers in these subtypes. This comprehensive approach aims to provide experimental evidence to support the pharmacological research and clinical application of Tryp and its derivatives.

## 2 Materials and methods

### 2.1 Cell culture and experimental protocols

HCT116 cells were purchased from Pricella (Wuhan, China), and SW480 cells were obtained from the Cell Resource Center, Institute of Basic Medical Sciences, Chinese Academy of Medical Sciences (Beijing, China). All cells were maintained in DMEM medium (Pricella, Wuhan, China) supplemented with 10% fetal bovine serum (Pricella, Wuhan, China) and 1% penicillin/streptomycin (Biosharp, Hefei, China). All cells were maintained in a humidified incubator set to 37°C with 5% CO_2_. Based on prior studies and preliminary experiments (Gao et al., 2021), cells were treated with various concentrations of Tryp (25, 50, 75, and 100 μM) or 8-Nitrotryp (0.5, 1, 1.5, 2, and 3 μM) for 24, 48, or 72 h.

TGF-β exhibits dual roles in CRC, acting as a tumor suppressor in early stages but promoting tumor progression in advanced disease (Morikawa et al., 2016). To model the TGF-β-enriched microenvironment of late-stage CRC, exogenous TGF-β stimulation was employed to investigate 8-Nitrotryp’s antagonism of oncogenic signaling. To recapitulate the TGF-β-enriched microenvironment characteristic of advanced CRC, cells were stimulated with exogenous recombinant human TGF-β1 (PeproTech, Cat. #100–21). Lyophilized TGF-β1 (10 μg/vial) was briefly centrifuged (12,000 rpm, 30 s) and reconstituted in 100 μL of sterile-filtered 10 mM citric acid (pH 3.0) to generate a 0.1 mg/mL stock solution. This stock was diluted in sterile 0.1% (w/v) bovine serum albumin (BSA; Sigma-Aldrich) in PBS to a working concentration of 10 μg/mL. For treatment, TGF-β1 was added to complete DMEM culture medium at a final concentration of 10 ng/mL, establishing conditions reflective of late-stage CRC stroma. Cells were incubated in this TGF-β1-enriched medium for the indicated experimental durations.

### 2.2 Chemicals and reagents

Tryp and 8-Nitrotryp were purchased from MedChemExpress (MCE, New Jersey, United States) with purities of 99.89% and 98.56%, respectively. Stock solutions were prepared using DMSO (Solarbio) as the solvent. Antibodies against E-cadherin, Slug, phospho-SMAD2, SMAD2, phospho-mTOR, mTOR, and goat anti-rabbit IgG-HRP were obtained from Cell Signaling Technology (Danvers, MA, United States). Antibodies against PI3K, phospho-AKT, and AKT were purchased from HUABIO (Hangzhou, China). β-Actin antibody and goat anti-mouse IgG conjugated with HRP were acquired from Proteintech (Wuhan, China).

### 2.3 Cell viability assay

Cell viability was assessed using the CCK-8 assay (Biosharp, Hefei, China). HCT116 and SW480 cells were plated in 96-well plates at a density of 5 × 10^3^ cells per well and exposed to different concentrations of Tryp or 8-Nitrotryp for 24, 48, or 72 h. CCK-8 solution was added to each well and incubated for 1 h at 37°C. Absorbance at 450 nm was measured using a microplate reader (BioTek, Winooski, VT, United States).

### 2.4 Colony formation assay

HCT116 and SW480 cells were plated in six-well plates at a density of 500 cells per well and exposed to varying concentrations of 8-Nitrotryp for 14 days. Colonies were fixed with 4% paraformaldehyde and stained with 0.1% crystal violet. Colonies containing >50 cells were observed and counted under a microscope.

### 2.5 Transwell migration assay

Transwell chambers (Corning, NY, United States) with 8-μm pores were used to assess cell migration. Cells (2 × 10^4^) in serum-free medium were placed in the upper chamber, while the lower chamber contained complete medium supplemented with 20% fetal bovine serum. After 48 h of incubation with different concentrations of 8-Nitrotryp, the migrated cells were fixed, stained, and counted under a microscope.

### 2.6 Mitochondrial membrane potential (MMP) analysis

MMP was evaluated using the JC-1 assay kit (Beyotime, Shanghai, China) following the manufacturer’s guidelines. Cells were treated with 8-Nitrotryp for 24 h and then stained with JC-1. Fluorescence intensity was measured using a microplate reader (BioTek, Winooski, VT, United States) with excitation/emission wavelengths of 485/530 nm (monomeric form) and 485/590 nm (aggregated form). Flow cytometry (BD Biosciences, San Jose, CA, United States) was used for additional validation.

### 2.7 Astral-DIA proteomics sequencing

The control group and a medium concentration (1 μM) drug-treated group of HCT116 cells, each consisting of three samples, were sent to Shanghai Majorbio Biopharm Technology Co., Ltd. for Astral-DIA proteomic sequencing. The sequencing results were analyzed using the Majorbio Cloud platform (cloud.majorbio.com), with a significance test threshold of *P* < 0.05. Proteins showing |fold change| >1.2 were considered differentially expressed. All differentially expressed proteins were annotated and functionally clustered using the GO database (Gene Ontology, http://geneontology.org/). Metabolic pathway analysis involving the differentially expressed proteins was conducted using the KEGG database (Kyoto Encyclopedia of Genes and Genomes, http://www.genome.jp/kegg/). Protein–protein interactions were analyzed using STRING v11.5.

### 2.8 Western blotting

Cells were lysed using ice-cold lysis buffer (Western & IP Lysis Buffer, Beyotime, China; Cat. #P0013) supplemented with freshly prepared protease inhibitor cocktail (Solarbio, China; Cat. #A8260) and phosphatase inhibitor cocktail (KeyGEN BioTECH, China; Cat. #KGP602). Lysates were centrifuged (12,000 × g, 15 min, 4°C), and the supernatants were collected. Protein concentrations were determined using the BCA assay kit (KeyGEN BioTECH, Nanjing, China). Equal amounts of protein (20 μg per lane) were separated by SDS-PAGE and electrotransferred onto PVDF membranes (Millipore, United States). Membranes were blocked with 5% (w/v) bovine serum albumin (BSA; Solarbio, China; Cat. #A8020) or non-fat dry milk (Solarbio; Cat. #D8340) in TBST for 1.5 h at room temperature with gentle agitation.

Blocked membranes were incubated with primary antibodies overnight at 4°C with constant shaking: E-cadherin (rabbit; Cell Signaling Technology [CST], United States; 1:1,000; Cat. #3195), Slug (rabbit; CST; 1:1,000; Cat. #9585), phospho-SMAD2 (rabbit; CST; 1:1,000; Cat. #3108), SMAD2 (rabbit; CST; 1:1,000; Cat. #5339), PI3K (mouse; HUABIO, China; 1:3,000; Cat. #HA722522), phospho-AKT (rabbit; HUABIO; 1:5,000; Cat. #ET1607-73), AKT (rabbit; HUABIO; 1:1,000; Cat. #WL0003b), phospho-mTOR (rabbit; CST; 1:1,000; Cat. #5536), mTOR (rabbit; CST; 1:1,000; Cat. #2972), and β-actin (mouse; Sanying Biotechnology, China; 1:20,000; Cat. #66009-1-Ig).

After washing, membranes were incubated for 1.5 h at room temperature by shaking with HRP-conjugated secondary antibodies: goat anti-rabbit IgG (1:2000; CST, United States; Cat. #7074) or goat anti-mouse IgG (1:5,000; Sanying, China; Cat. #SA00001-1). Protein bands were detected using an ECL kit (NCM Biotech, China; Cat. #P10300) and imaged using an Amersham Imager 600 system (GE Healthcare, United States). Band intensity was quantified using ImageJ software (NIH, United States), and protein expression levels were normalized to β-actin.

### 2.9 Statistical analysis

Graphs were constructed using GraphPad Prism 8.0 (GraphPad Software, San Diego, CA, United States). Data are presented as mean ± SD. Two-group comparisons used *t*-tests, multi-group comparisons used one-way ANOVA, and all analyses were based on ≥3 independent experiments with statistical significance defined as a p-value below 0.05.

## 3 Results

### 3.1 8-Nitrotryp inhibits proliferation of HCT116 and SW480 cells

**FIGURE 1:**
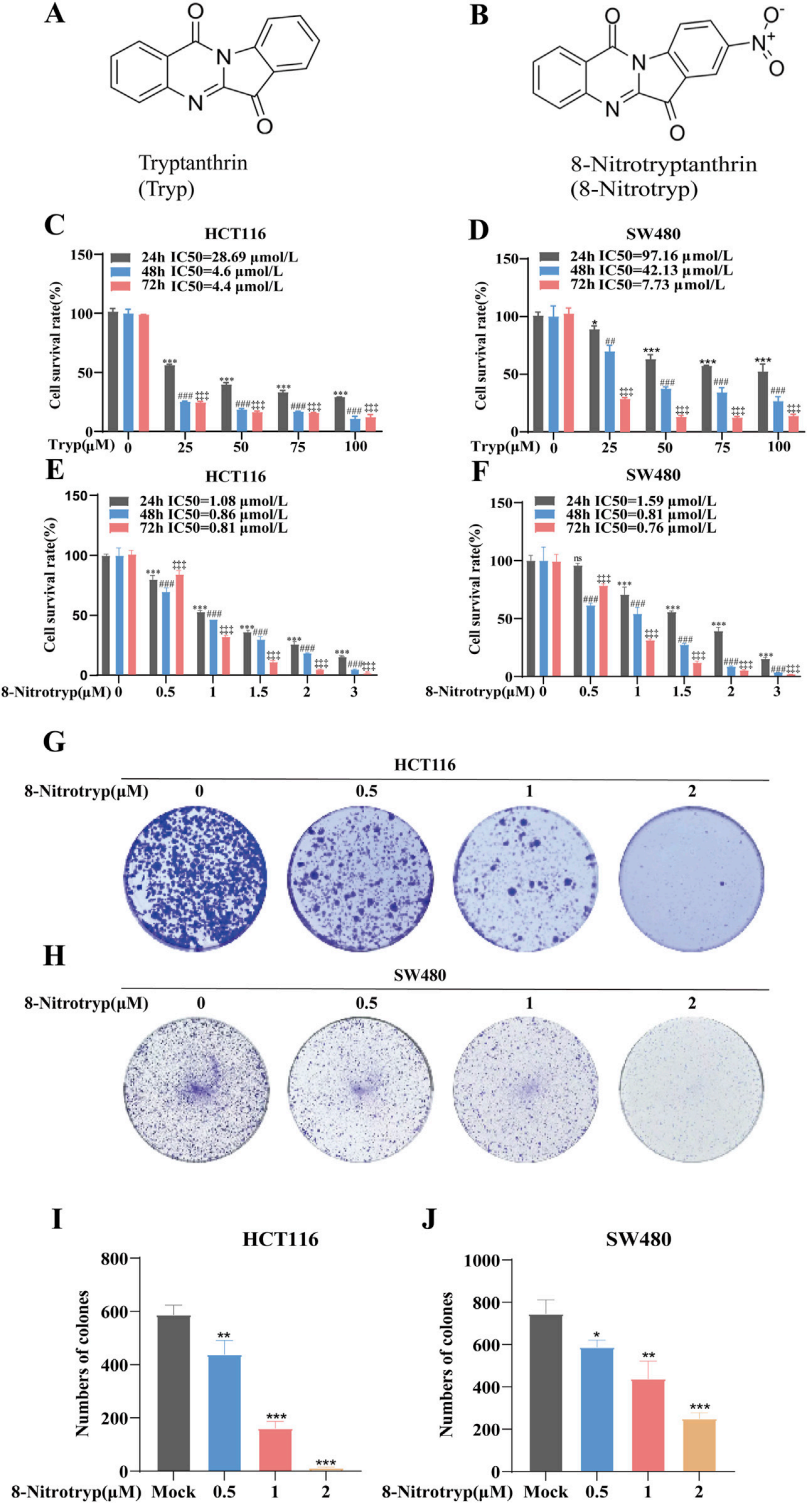
Tryptanthrin (Tryp) and 8-nitrotryptanthrin (8-Nitrotryp) inhibit CRC cell growth and colony formation. **(A,B)** Chemical structures of Tryp and 8-Nitrotryp. **(C–F)** Inhibitory effects of Tryp and 8-Nitrotryp on the proliferation of HCT116 and SW480 cells. Cells were treated with various concentrations of Tryp (25, 50, 75, and 100 μM) or 8-Nitrotryp (0.5, 1, 1.5, 2, and 3 μM) for 24 h, 48 h, and 72 h. Cell viability was assessed using CCK-8 assay. **(G,H)** Representative images of colony formation assays for HCT116 and SW480 cells. Cells were treated with low, medium, and high concentrations (0.5, 1, and 2 μM) of 8-Nitrotryp for 15 days. **(I,J)** Quantitative analysis of colony numbers from colony formation assays. All experiments were performed independently in triplicate. #*P* < 0.05, ##*P* < 0.01, and ###*P* < 0.001 compared with control (24 h); #*P* < 0.05, ##*P* < 0.01, and ###*P* < 0.001 compared to control (48 h); #*P* < 0.05, ##*P* < 0.01, and ###*P* < 0.001 compared to control (72 h).

Figures 1A,B illustrate the chemical structures of Tryp and 8-Nitrotryp, respectively. Initially, two kinds of cells were treated with different concentrations of Tryp (25, 50, 75, and 100 μM) and 8-Nitrotryp (0.5, 1, 1.5, 2, and 3 μM) for 24, 48, and 72 h. The results demonstrated that both Tryp and 8-Nitrotryp significantly inhibited the proliferation of CRC cells in a concentration-dependent manner, with 8-Nitrotryp exhibiting a markedly superior inhibitory effect on cell proliferation compared to its parent compound, Tryp (Figures 1C–F). The IC50 values of 8-Nitrotryp were as follows: HCT116—1.08 μM (24 h), 0.86 μM (48 h), and 0.81 μM (72 h); SW480—1.59 μM (24 h), 0.81 μM (48 h), and 0.76 μM (72 h). Consequently, subsequent experiments on HCT116 and SW480 cells were conducted using low, medium, and high concentrations of 8-Nitrotryp (0.5, 1, and 2 μM). According to the results of the colony formation assay, 8-Nitrotryp compared to the control group significantly reduced the number of colonies formed by HCT116 and SW480 cells in a concentration-dependent manner (Figures 1G–J).

### 3.2 8-Nitrotryp suppresses migration of HCT116 and SW480 cells

**FIGURE 2:**
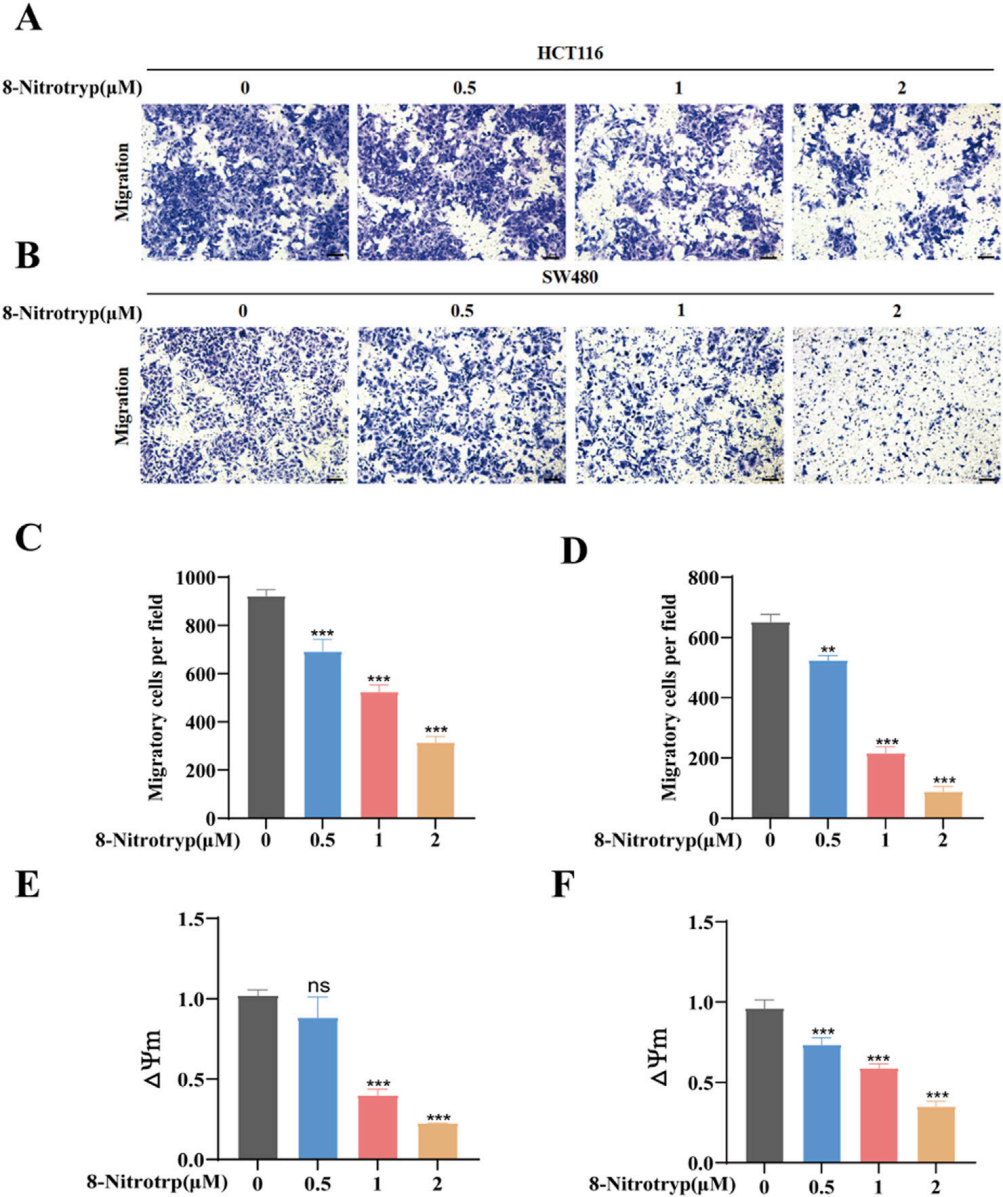
8-Nitrotryp inhibits CRC cell migration and reduces MMP. **(A,B)** Representative images (10× magnification) of HCT116 and SW480 cell migration; scale bars represent 100 μm. **(C,D)** Quantitative analysis of migrated HCT116 and SW480 cells after treatment with different concentrations (0.5, 1, and 2 μM) of 8-Nitrotryp for 48 h. **(E,F)** Quantitative analysis of MMP in HCT116 and SW480 cells after treatment with different concentrations (0.5, 1, and 2 μM) of 8-Nitrotryp for 24 h. △Ψm representing MMP, which is the ratio of red to green fluorescence. All experiments were performed independently in triplicate. *P < 0.05, **P < 0.01, and ***P < 0.001 compared to control.

To assess the impact of 8-Nitrotryp on the migratory capabilities of CRC cells, we conducted Transwell migration assays on two cell lines: HCT116 and SW480. As depicted in Figures 2A–D, the cells were treated with 0.5 μM, 1 μM, and 2 μM of the drug for 48 h. Cell migration to the lower chamber decreased dose-dependently (Figures 2A–D). These results confirm that 8-Nitrotryp significantly inhibits the migration of CRC cells.

### 3.3 8-Nitrotryp induces apoptosis via MMP reduction

MMP was assessed in 8-Nitrotryp-treated cells using JC-1 staining. As illustrated in Figures 2E,F, MMP decreased with increasing concentrations of the drug. These findings indicate that 8-Nitrotryp can reduce MMP, thereby inducing early apoptosis in CRC cells.

### 3.4 Proteomic profiling

#### 3.4.1 Proteomic profiling and differential protein expression in HCT116 cells

**FIGURE 3:**
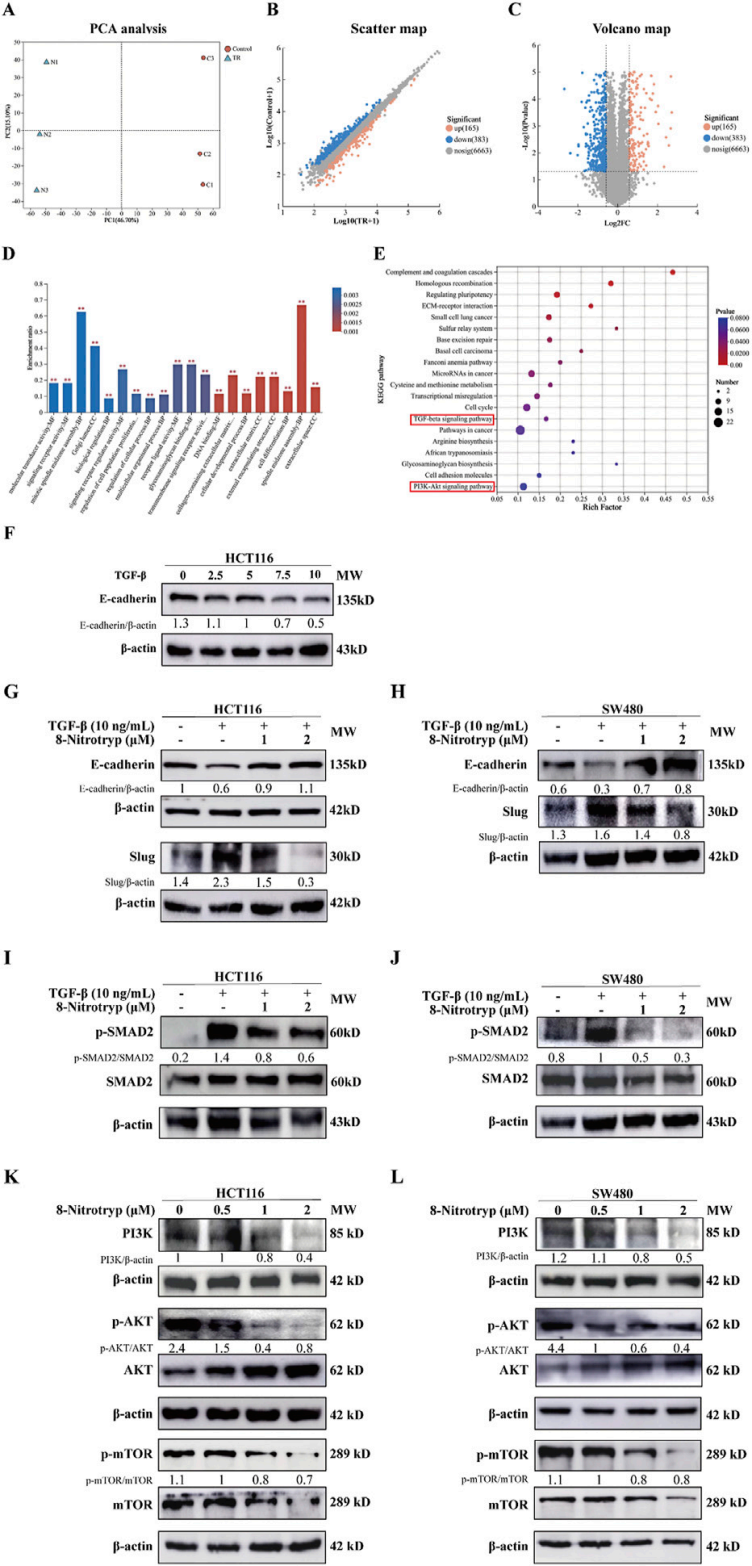
8-Nitrotryp inhibits the TGF-β/SMAD and PI3K/AKT/mTOR pathways. **(A–C)** Astral-DIA proteomics sequencing of HCT116 cells revealed significant differences between the treatment (1 μM) and control groups. Scatter plots and volcano plots showed that 165 proteins were significantly upregulated, 383 were significantly downregulated, and 6,663 proteins showed no significant differences. **(D)** Gene ontology (GO) enrichment analysis of Astral-DIA sequencing results revealed significant differences between the treatment (1 μM) and control groups. **(E)** Kyoto Encyclopedia of Genes and Genomes (KEGG) enrichment analysis of Astral-DIA sequencing results revealed that the TGF-β/SMAD and PI3K/AKT/mTOR pathways were involved in the anti-HCT116 and anti-SW480 cell processes of 8-Nitrotryp. **(F)** Western blotting analysis of the effects of different concentrations of TGF-β1 (2.5, 5, 7.5, and 10 ng/mL) on E-cadherin expression in HCT116 cells **(G,H)** Western blotting analysis of the effects of 8-Nitrotryp on the expression of EMT-related proteins. HCT116 and SW480 cells were treated with 10 ng/mL TGF-β1 and different concentrations of 8-Nitrotryp (1 μM and 2 μM) for 48 h; β-actin was used as reference. **(I,J)** Western blotting analysis of the effects of 8-Nitrotryp on SMAD2 protein expression. HCT116 and SW480 cells were treated with 10 ng/mL TGF-β1 and different concentrations of 8-Nitrotryp (1 μM and 2 μM) for 48 h; β-actin was used as reference. **(K,L)** Western blotting analysis of the effects of 8-Nitrotryp on the expression of key proteins in the PI3K/AKT/mTOR pathway. HCT116 and SW480 cells were treated with different concentrations of 8-Nitrotryp (0.5, 1, and 2 μM) for 24 h; β-actin was used as reference.

To further explore the mechanisms by which 8-Nitrotryp exerts its anti-CRC effects, we selected HCT116 cells, which showed a more pronounced inhibitory effect after drug treatment. We conducted Astral-DIA proteomics analysis on three control groups and three groups treated with a moderate concentration of the drug (1 μM). The results of principal component analysis (PCA) for the two sample groups are shown in Figure 3A. Intra-group protein profiles showed high reproducibility. The control and treatment groups are clearly separated, with significant differences between the two. This suggests that 8-Nitrotryp treatment has a notable impact on the proteome of HCT116 cells. Visualization of differential proteins between the treated and control groups revealed that 165 proteins were significantly upregulated, 383 were significantly downregulated after treatment with 8-Nitrotryp, and 6,663 proteins showed no significant difference (Figures 3B,C). The results of GO enrichment analysis for differentially expressed proteins are presented in Figure 3D. The proteins are distributed across three categories: cellular component (CC), biological process (BP), and molecular function (MF). GO enrichment analysis revealed that 8-Nitrotryp significantly improved functions such as cell differentiation, cellular developmental processes, and DNA binding. As shown in Figure 3E, KEGG enrichment analysis further highlights significant alterations in the TGF-β signaling pathway. Other pathways related to tumor cell functions, including the PI3K-AKT pathway, were also identified. The prioritization of TGF-β/SMAD2 and PI3K/AKT pathways in this study stems from their coherent enrichment trends in differential proteins, which align with established CRC phenotypes. Experimental validation confirmed that 8-Nitrotryp concurrently suppresses both pathways, mirroring phenotypic outcomes (e.g., reduced migration and increased apoptosis), suggesting their synergistic roles in CRC progression. Based on these findings, we proceeded with Western blotting to examine molecular markers associated with these two pathways.

#### 3.4.2 Effects of 8-Nitrotryp on EMT marker expression in CRC cells

To establish an EMT model, HCT116 cells were treated with TGF-β1 (0, 2.5, 5, 7.5, and 10 ng/mL) for 48 h. As illustrated in Figure 3F, the expression of the EMT-associated protein E-cadherin gradually declined as TGF-β1 concentration increased. Treatment with 10 ng/mL TGF-β1 induced a stable reduction in E-cadherin expression. Consequently, subsequent experiments included a control group, a 10 ng/mL TGF-β1 group, and 8-Nitrotryp-treated groups with different concentrations (0.5 μM, 1 μM, and 2 μM). The effects of 8-Nitrotryp on E-cadherin and Slug expression were analyzed.

As shown in Figures 3G,H, TGF-β1 treatment reduced E-cadherin expression and increased Slug expression. However, the addition of 8-Nitrotryp reversed these trends, with E-cadherin expression increasing and Slug expression decreasing. The effects became more pronounced with higher concentrations of 8-Nitrotryp. These results were consistent in both cell lines, suggesting that 8-Nitrotryp inhibits EMT progression in CRC cells.

#### 3.4.3 Inhibition of TGF-β/SMAD and PI3K/AKT/mTOR pathways by 8-Nitrotryp

To explore the mechanism by which 8-Nitrotryp mitigates EMT, key proteins in the canonical SMAD and non-canonical pathways were analyzed. In the SMAD pathway (Figures 3I,J), TGF-β1 treatment increased SMAD2 phosphorylation in both cell lines. This effect was attenuated by 8-Nitrotryp in a dose-dependent manner. The downregulation of SMAD2 phosphorylation subsequently influenced the expression of EMT-related proteins. Therefore, 8-Nitrotryp suppresses EMT in HCT116 and SW480 cells by inhibiting SMAD2 phosphorylation, thereby reducing tumor metastasis.

**FIGURE 4:**
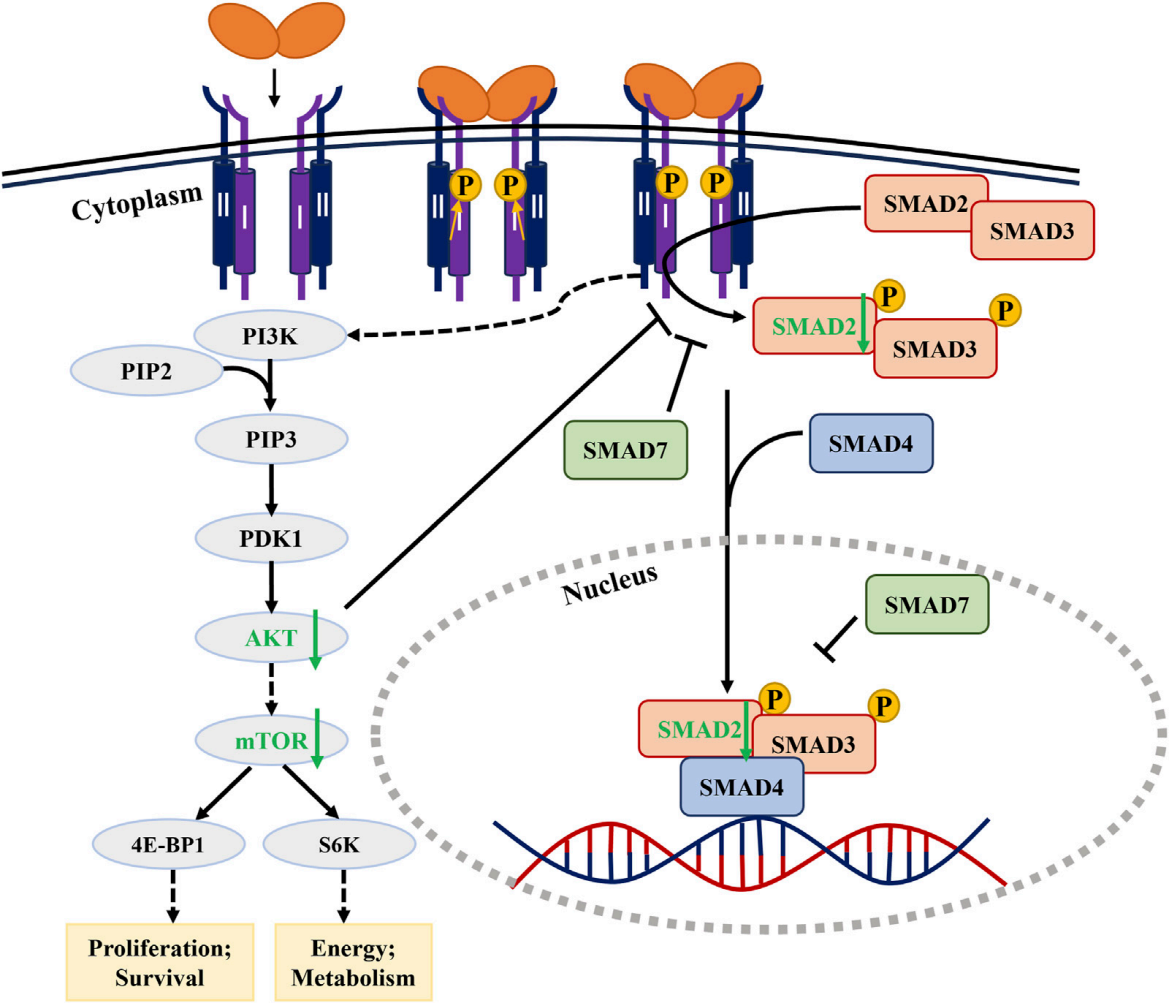
Signaling pathway diagram of 8-Nitrotryp inhibiting CRC through TGF-β/SMAD and PI3K/AKT/mTOR pathways.

In the non-canonical PI3K/AKT/mTOR pathway (Figures 3K,L), 8-Nitrotryp dose-dependently suppressed PI3K and mTOR expression in both cell lines. For PI3K and mTOR, the inhibitory effects were concentration-dependent. AKT expression followed a consistent trend in SW480 cells. In HCT116 cells, AKT expression showed a biphasic response—initial increase followed by decrease—at higher concentrations. Despite this fluctuation, AKT levels remained significantly lower than those in the control group. These findings indicate that 8-Nitrotryp suppresses protein synthesis and inhibits tumor metastasis in HCT116 and SW480 cells by downregulating PI3K, mTOR, and AKT expression. The specific pathway diagram is shown in Figure 4.

In summary, 8-Nitrotryp inhibits the biological activity of CRC HCT116 and SW480 cells by suppressing the TGF-β/SMAD and PI3K/AKT/mTOR pathways.

## 4 Discussion

### 4.1 Challenges in CRC treatment and the potential of 8-Nitrotryp

CRC treatment options include surgical resection, chemotherapy, radiotherapy, immunotherapy, and targeted therapy. However, effective therapeutic strategies are still lacking in the clinic, and the disease is often associated with issues such as drug resistance, metastasis, recurrence, poor prognosis, and significant individual variability (Kanas et al., 2012; Barlow et al., 2009). In CRC, EMT not only endows cancer cells with invasive and migratory abilities but is also closely related to chemotherapy resistance and immune escape (Li et al., 2016). Therefore, targeting the molecular mechanisms of EMT is an important direction for CRC therapy. This study investigates the suppressive effects of 8-Nitrotryp on the malignant characteristics of HCT116 and SW480 cells and explores the mechanism by which 8-Nitrotryp suppresses tumor cell EMT progression, providing insights for CRC treatment.

### 4.2 The role of 8-Nitrotryp and Tryp in inhibiting the malignant phenotype of CRC

Modern pharmacological studies have demonstrated that tryptamine and its derivatives exhibit various pharmacological activities, including antitumor and antibacterial effects (Sun et al., 2023). In this study, the effects of tryptamine and 8-Nitrotryp on the proliferation, migration, and apoptosis of two CRC cell lines were evaluated. The results confirmed that both 8-Nitrotryp and tryptamine inhibited the malignant phenotype of HCT116 and SW480 cells in a manner dependent on both concentration and time. Additionally, previous studies have reported that nitro-substituted tryptamine derivatives possess inhibitory activity against various tumor cells, with some derivatives exhibiting significantly higher activity than the parent compound (Khan et al., 2025). For instance, nitro-substituted tryptamine derivatives demonstrated an IC50 value of 1.0 mmol·L^−1^ against breast cancer cells MCF7/ADR, which is significantly more effective than the parent compound (Sharma et al., 2002; Liu et al., 2022). Structure–activity relationship (SAR) analysis revealed that nitro-substitution at the C8 position of Tryp significantly enhances its antiproliferative potency. Based on the experimental results of IC50 values in this study, it was observed that 8-Nitrotryp exhibits several-fold higher antiproliferative activity than Tryp across both cell lines, consistent with prior research showing that nitro groups improve target binding through enhanced electron-withdrawing effects (Gao et al., 2021). Our findings reveal that 8-Nitrotryp is significantly more effective than its parent compound, Tryp, in inhibiting the growth of HCT116 and SW480 cells. This enhanced activity may be attributed to the nitro group modification, which could potentially improve the compound’s cellular uptake or target binding affinity. In summary, both 8-Nitrotryp and tryptamine suppress the malignant phenotype of CRC cells through multiple mechanisms. These effects are characterized by a significant inhibition of cell proliferation, leading to reduced tumor cell growth rates, attenuation of cell migration ability that prevents the spread of cancer cells to surrounding tissues, and the induction of early apoptosis, accelerating the clearance of cancer cells. Together, these actions effectively impede key processes involved in tumor progression.

### 4.3 8-Nitrotryp inhibits TGF-β1-induced EMT process, suppressing tumor metastasis in CRC cells

Preventing tumor metastasis is a crucial step in controlling the malignant progression of cancer. EMT plays a pivotal role in cancer metastasis (Thiery et al., 2009). The reduced expression of the epithelial marker E-cadherin marks the initiation of the EMT process, indicating the potential for tumor metastasis (Wong et al., 2018). Our research revealed that TGF-β1 induces a concentration-dependent downregulation of E-cadherin, thereby initiating EMT and upregulating the mesenchymal marker Slug. Furthermore, treatment with 8-Nitrotryp resulted in the upregulation of E-cadherin and downregulation of Slug, indicating that 8-Nitrotryp can inhibit the TGF-β1-induced EMT process in CRC and thereby suppress tumor metastasis. This is consistent with the results of the Transwell migration assay in this study. It was demonstrated that 8-Nitrotryp suppresses TGF-β1-induced EMT, thereby reducing the invasive and migratory abilities of CRC cells. Consequently, tumor metastasis was effectively prevented and inhibited.

### 4.4 8-Nitrotryp suppresses CRC progression and metastasis by inhibiting the TGF-β/SMAD signaling pathway

In CRC, EMT not only endows cancer cells with invasive and migratory abilities but also drives tumor heterogeneity and drug resistance through interactions with multiple signaling pathways. Unlike single-target TGF-β inhibitors (e.g., galunisertib), which face challenges due to compensatory pathway activation, 8-Nitrotryp’s dual inhibition of the TGF-β/SMAD and PI3K/AKT/mTOR pathways disrupts crosstalk mechanisms driving EMT and chemoresistance (Ungefroren, 2021). This multi-target approach aligns with recent trends in CRC therapy to overcome adaptive resistance. Based on KEGG and GO enrichment analysis from Astral-DIA proteomics, the TGF-β pathway was identified for further investigation. In advanced stages of cancer, CRC cells release TGF-β, which inhibits the differentiation of immature T cells into Th1 cells, encourages their conversion into Treg subsets, and impairs the antigen-presenting activity of dendritic cells, facilitating immune evasion. As the tumor progresses, mutations in TGF-β receptors or downstream SMAD genes accumulate, reducing its inhibitory effects. Additionally, TGF-β signaling promotes EMT, a process that transforms polarized epithelial cells into motile mesenchymal cells, thereby conferring invasive and migratory capabilities. Studies have also reported that various oncogenes can facilitate CRC progression by enhancing SMAD2/3 activation or expression and activating the TGF-β pathway (Akhurst and Hata, 2012; Wu et al., 2021; Di et al., 2023). Inhibiting the activation of SMADs proteins can prevent tumor progression (Ji et al., 2015). The results of this study demonstrated that 8-Nitrotryp downregulated SMAD2 phosphorylation induced by TGF-β1, upregulated E-cadherin expression, and downregulated Slug expression. These effects inhibited EMT, reducing the invasive and migratory abilities of CRC cells. Therefore, the inhibition of the TGF-β/SMAD pathway is identified as a key mechanism by which 8-Nitrotryp suppresses CRC metastasis.

### 4.5 8-Nitrotryp inhibits CRC cell proliferation and survival by suppressing the PI3K/AKT/mTOR pathway

Notably, the simultaneous inhibition of both TGF-β/SMAD and PI3K/AKT/mTOR pathways by 8-Nitrotryp addresses a critical limitation of conventional monotherapies—the inability to block compensatory crosstalk between oncogenic signaling axes. Beyond the canonical SMAD pathway, TGF-β receptor complexes can additionally relay signals through alternative molecules, including tumor necrosis factor receptor-associated factor 4 (TRAF4), tumor necrosis factor receptor-associated factor 6 (TRAF6), transforming growth factor-β-activated kinase 1 (TAK1), p38 mitogen-activated protein kinase (p38 MAPK), Ras homolog family members (RHO), PI3K/AKT, extracellular signal-regulated kinase (ERK), c-Jun N-terminal kinase (JNK), and nuclear factor kappa-B (NF-κB). These pathways are indirectly involved in apoptosis, EMT, migration, proliferation, differentiation, and matrix formation (Liu et al., 2023; Fruman et al., 2017). The PI3K/AKT/mTOR pathway is hyperactivated in >40% of CRC cases, and its inhibition by agents like pictilisib has shown limited clinical efficacy due to feedback activation of parallel pathways (Cancer Genome Atlas Network, 2012). In contrast, 8-Nitrotryp’s concomitant suppression of TGF-β/SMAD signaling may prevent such compensatory mechanisms, as evidenced by sustained MMP reduction and apoptosis induction (Figures 2E,F). The inhibition of this pathway can lead to reduced cell proliferation, survival, and metabolism, making it an attractive target for cancer therapy (Huang et al., 2017). Our findings demonstrate that 8-Nitrotryp inhibits PI3K protein expression and the phosphorylation of its downstream key proteins AKT and mTOR. AKT is one of the primary downstream effectors of PI3K. Its pleckstrin homology (PH) domain specifically binds to phosphatidylinositol bisphosphate (PIP2) and phosphatidylinositol-3,4,5-triphosphate (PIP3), enabling AKT localization to the cell membrane. Subsequently, the kinase domain transfers a phosphate group from ATP to the substrate’s threonine, leading to phosphorylation. Mutation at E17K in the PH domain of AKT1 mediates sustained activation of AKT1 and its downstream pathways, promoting tumor formation (Saxton and Sabatini, 2017). mTORC1, located downstream of AKT, drives tumorigenesis by enhancing the transcription of proto-oncogenes and promoting angiogenesis. Its direct downstream substrate, S6 kinase, facilitates metabolic reprogramming by enhancing glycolysis and the biosynthesis of proteins, lipids, and nucleic acids. Another downstream substrate, eukaryotic initiation factor 4E-binding protein 1 (4E-BP1), promotes tumor cell proliferation and survival by regulating the transcription initiation complex eukaryotic initiation factor 4F (EIF4F) (Yang et al., 2022). Based on previous experimental results, 8-Nitrotryp significantly reduces MMP, inhibits cancer cell proliferation and induces early apoptosis (Figures 2E,F). This indicates that it suppresses the expression of mTOR and AKT, thereby inhibiting protein synthesis, energy metabolism, and other related physiological activities in CRC cells HCT116 and SW480. Consequently, it induces early apoptosis and inhibits cancer cell proliferation. Additionally, the observed decrease in MMP with increasing drug concentration further supports the anti-CRC potential of 8-Nitrotryp. Therefore, the PI3K/AKT/mTOR pathway is inhibited, representing another key mechanism by which 8-Nitrotryp exerts its anti-CRC effects.

### 4.6 The prospects and challenges of 8-Nitrotryp in CRC treatment

In this study, 8-Nitrotryp demonstrated significant antitumor effects by inhibiting CRC cell proliferation and migration and inducing early apoptosis through suppression of the TGF-β/SMAD and PI3K/AKT/mTOR pathways. The dual-pathway targeting capability of 8-Nitrotryp positions it as a promising candidate for combination therapies. For instance, synergism with immune checkpoint inhibitors could be explored, given TGF-β's role in immunosuppression and PI3K’s involvement in T-cell exhaustion. However, challenges such as bioavailability optimization and toxicity profiling require further investigation (Gaggianesi et al., 2022). However, several limitations remain. First, the research was primarily conducted at the cellular level; animal studies are needed to validate its efficacy and safety. Second, while the study highlighted its effects on CRC, its inhibitory activity against other types of tumors requires further investigation. Future research should expand the scope of its application and explore the clinical potential of 8-Nitrotryp.

## 5 Conclusion

This study demonstrates that 8-Nitrotryp inhibits colorectal cancer progression through dual suppression of the TGF-β/SMAD and PI3K/AKT/mTOR pathways. By reducing SMAD2 phosphorylation, it suppresses EMT-mediated invasion and metastasis while concurrently inhibiting proliferative signaling to induce apoptosis. These findings establish 8-Nitrotryp as a promising therapeutic candidate, warranting further evaluation through nanoparticle-based delivery systems to enhance bioavailability, validation across heterogeneous CRC cell lines (including mesenchymal and chemoresistant subtypes), efficacy assessment in patient-derived xenograft and metastatic animal models, and exploration of clinical applications for TGF-β/PI3K-hyperactivated CRC. Such translational studies will bridge mechanistic insights into therapeutic implementation, addressing unmet needs in advanced CRC treatment.

## Data Availability

The raw data supporting the conclusions of this article will be made available by the authors, without undue reservation.

## Abbreviations

BP, biological process; CC, cellular component; CRC, colorectal cancer; EMT, epithelial–mesenchymal transition; MMP, mitochondrial membrane potential; MF, molecular function; Tryp, tryptanthrin; 8-Nitrotryp, 8-nitrotryptanthrin.
